# Mitochondria-targeted Triphenylamine Derivatives Activatable by Two-Photon Excitation for Triggering and Imaging Cell Apoptosis

**DOI:** 10.1038/srep21458

**Published:** 2016-03-07

**Authors:** Rahima Chennoufi, Houcine Bougherara, Nathalie Gagey-Eilstein, Blaise Dumat, Etienne Henry, Frédéric Subra, Stéphanie Bury-Moné, Florence Mahuteau-Betzer, Patrick Tauc, Marie-Paule Teulade-Fichou, Eric Deprez

**Affiliations:** 1 LBPA, CNRS UMR8113, IDA FR3242, ENS Cachan, Université Paris-Saclay, F-94235 Cachan, France; 2 Chemistry, Modeling and Imaging for Biology, UMR9187-U1196, Institut Curie, Centre universitaire, 91405 Orsay, France

## Abstract

Photodynamic therapy (PDT) leads to cell death by using a combination of a photosensitizer and an external light source for the production of lethal doses of reactive oxygen species (ROS). Since a major limitation of PDT is the poor penetration of UV-visible light in tissues, there is a strong need for organic compounds whose activation is compatible with near-infrared excitation. Triphenylamines (TPAs) are fluorescent compounds, recently shown to efficiently trigger cell death upon visible light irradiation (458 nm), however outside the so-called optical/therapeutic window. Here, we report that TPAs target cytosolic organelles of living cells, mainly mitochondria, triggering a fast apoptosis upon two-photon excitation, thanks to their large two-photon absorption cross-sections in the 760–860 nm range. Direct ROS imaging in the cell context upon multiphoton excitation of TPA and three-color flow cytometric analysis showing phosphatidylserine externalization indicate that TPA photoactivation is primarily related to the mitochondrial apoptotic pathway via ROS production, although significant differences in the time courses of cell death-related events were observed, depending on the compound. TPAs represent a new class of water-soluble organic photosensitizers compatible with direct two-photon excitation, enabling simultaneous multiphoton fluorescence imaging of cell death since a concomitant subcellular TPA re-distribution occurs in apoptotic cells.

Photodynamic therapy (PDT) leads to cell death by using a combination of a photosensitizer under light excitation for the production of lethal doses of the so-called Reactive Oxygen Species (ROS)^1^,^2^. Porphyrins and chlorins, exemplified by the well-known porfimer sodium (Photofrin^®^) and Temoporfin (*meta*-tetra(hydroxyl-phenyl)chlorin, Foscan^®^), respectively, are among the most potent classes of compounds used in PDT of malignancies. Significant enhancements of PDT efficacy can be achieved using combination treatments in which the effect of a photosensitizer can be improved by another chemical compound specifically targeting and inhibiting cellular pathways, such as for instance the survivin pathway^3^ or the proteasome-dependent protein degradation^4^, or selectively enhancing accumulation of the PDT compound within tumor cells^5^.

Another concern for PDT improvement is related to the pathway of photosensitizer excitation. Most of the standard PDT compounds developed so far are characterized by one-photon activation wavelengths in the red part of the visible spectrum, typically in the 630–690 nm range in the so-called therapeutic window, to optimize light penetration by minimizing both light scattering and absorption at the tissue level^1^. Recently, visible/near-infrared (NIR) one-photon excitation (680–700 nm) of phthalocyanine dye coupled to a monoclonal antibody was used for photoimmunotherapy^6^. However, the use of NIR light for direct sensitization in PDT remains limited in the one-photon excitation mode beyond 750 nm due to insufficient energy for singlet oxygen production^7^. Recently, an alternative (indirect) approach was developed based on up-converting (NIR-to-visible) nanoparticles loaded with standard PDT compounds activatable by visible light leading to ROS formation under NIR irradiation at 980 nm^8^. The up-conversion of NIR light by endogenous biomolecules (*e.g.* 2^nd^ harmonic generation) was also used for the one-photon activation of chlorin e6^9^. Consequently, there is a need for compounds whose activation occurs directly at wavelengths with optimal tissue penetration, typically in the NIR region of the spectrum (750–950 nm). This two-photon therapeutic window ensures a deeper penetration of light in tissue. Outside this range, visible light is strongly scattered or absorbed by redox co-factors or endogenous proteins, mainly hemoproteins, while NIR light absorption by water is high above 950 nm. Besides the deeper penetration, two-photon PDT could lead to a higher degree of spatial selectivity due to the quadratic dependence of excitation probability on light intensity. Current PDT compounds are poorly compatible with two-photon excitation. For instance, photofrin^®^ or Visudyne^®^ may induce cell death but requires very high light doses (6300–11300 J/cm^2^ and 1700 J/cm^2^, respectively) due to a low two-photon absoption cross-section (σ^2^)^10^,^11^. Further progress have been achieved with porphyrin derivatives and porphyrin dimers characterized by higher σ^2^ values^12^,^13^ or porphyrin core covalently linked to bis(diphenylamino)distyrylbenzene groups that display suitable two-photon absorption properties^7^. An indirect approach involves either energy transfer-mediated activation of a standard (one-photon) PDT compound (acceptor) by a two-photon absorbing dye acting as a donor within the same nanoparticle^14^ or a plasmon-mediated enhancement of two-photon excitation of the photosensitizer (*e.g.* the case of porphyrin in gold nanoparticles)^15^.

Based on the above-mentioned considerations, there is a great demand for new small organic PDT compounds compatible with direct two-photon excitation, characterized by easy synthesis, high chemical stability and satisfying the criteria of biocompatibility^16^. The triphenylamine compounds (TPA) have 2 or 3 vinyl branches with pyridinium (Py) or N-methyl benzimidazolium (Bzim) groups^17^,^18^. They were initially designed for nonlinear (two-photon) absorption due to their octupolar organization and display high chemical- and photo-stability as well as good water solubility and cellular uptake^17^,^18^,^19^. They bind to the DNA minor groove and their binding leads to a dramatic enhancement of the fluorescence signal with a maximum emission wavelength comprised between 555 and 685 nm, depending on the compound^17^,^18^,^19^. Accordingly, TPA-treated fixed cells display nuclear staining with excellent contrast, using one- or two-photon microscopy^17^,^18^,^19^,^20^. Recently, we have shown that fate and behaviour of TPAs were dramatically different in living cells^20^. They did not directly reach the nucleus but remained localized in the cytoplasm of living cells until photoexcitation. Upon visible light irradiation (450 nm), TPAs escaped from the cytoplasm and rapidly re-localized to the nucleus. Concomitant with this translocation, TPAs were able to induce rapid and massive cell death of cells in the submicromolar/low micromolar concentration range. Conversely, without an external/artificial source of excitation, TPAs remained in cytoplasm and displayed negligible dark or daylight cytotoxicities below 30 μM. Although TPAs display interesting properties in living cells, their photoactivation in the visible region of the spectrum requires wavelengths (<510 nm) far from the therapteutic window. We have previously demonstrated that TPAs are suitable for two-photon imaging of fixed cell nuclei^18^,^19^, however the effect of their two-photon excitation using NIR light was never addressed in the context of living cells and is of particular interest for therapeutic applications. Here, we address the question whether the overall process (nuclear translocation and cell death) could be compatible with two-photon excitation, taking into account the good two-photon absorption cross-sections of TPAs with σ^2^ values comprised between 250 and 1080 GM (1GM = 10^−50^cm^4^.s.photon^−1^.molecule^−1^)^17^,^18^,^19^. For the first time, we found that TP2Py and TP3Bzim were both compatible with photo-induced cell death via a two-photon excitation process and simultaneous multiphoton imaging of cell death. We also addressed in this study the mechanism of cell death involved upon photoactivation of TPAs in relationship with their intial subcellular localization. TPAs primarily trigger the mitochondrial apoptotic pathway even though a more in-depth temporal study of TP2Py/TP3Bzim, in terms of target organelles and cell death mechanism (apoptosis versus necrosis), indicates that subtle differences exist regarding their respective subcellular fates and photo-induced effects.

## Results

**Figure 1:**
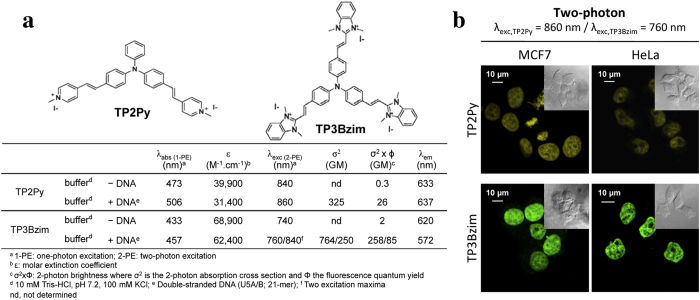
Structures, optical properties (**a**) and subcellular localization of TPAs in fixed cells (**b**). Two-photon imaging was performed with fixed MCF7 (left) or HeLa (right) cells treated with TP2Py (top) or TP3Bzim (bottom). Cells were first pre-incubated with 2 μM TPA for 2 h and then fixed with 4% paraformaldehyde. The emission slit settings for two-photon imaging was 560–720 nm and 530–690 nm for TP2Py and TP3Bzim, respectively. Insets: corresponding DIC (differential interference contrast) transmission images.

The characteristic λ_abs_, λ_em_ and σ^2^ values for TP2Py and TP3Bzim, two members of the TPA family (2- and 3-branch compounds, respectively) and used throughout the present study, are reported in Fig. 1a. The DNA-binding properties of TPAs combined to their large two-photon absorption cross-sections were initially considered as promising for multiphoton fluorescence imaging of the nucleus. Accordingly, when fixed using paraformaldehyde, TPA-treated cells such as MCF7 (breast cancer) or HeLa (cervical cancer) clearly showed a nuclear localization of TPAs with bright staining in two-photon imaging (Fig. 1b). This is in accordance with previous results obtained using fixed MCF7 or HeLa cells studied by one-photon microscopy^20^ or using fixed MRC5 cells studied by multiphoton microscopy^18^,^19^.

### Analysis of the cytoplasm-nucleus translocation of TPAs and concomitant cell death upon two-photon excitation

**Figure 2:**
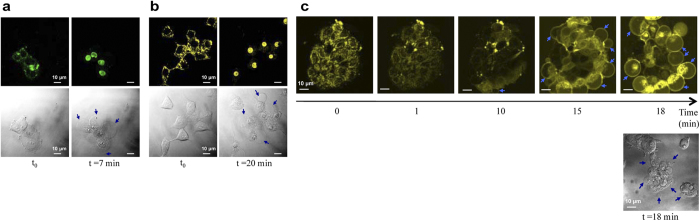
Death of MCF7 cells induced by two-photon excitation of TPAs. Living MCF7 cells pre-treated with TP3Bzim (**a**) or TP2Py (**b**) were exposed to two-photon illumination using a pulsed IR laser as an excitation source (irradiance, 1.25 W.cm^−2^). λ_exc_ = 760 and 860 nm for TP3Bzim (emission slit: 530–690 nm) and TP2Py (emission slit: 560–720 nm), respectively. Left, initial observation (t = 0). Right, observation after an illumination time of 7 min (TP3Bzim) or 20 min (TP2Py). Corresponding DIC transmission images illustrating membrane blebbing (blue arrows) are shown below fluorescence images. The time of illumination was calculated by taking into consideration the z-stack as indicated in Methods. (**c**) Two-photon fluorescence images showing the time-dependent re-localization of TP2Py (cytoplasm −>nucleus) as well as the formation of blebs (blue arrows) upon excitation at 860 nm (bottom: DIC transmission image).

We next investigated the effect of two-photon excitation of TPAs in living cells. Living MCF7 cells pre-treated with TPAs were exposed to NIR excitation and simultaneously observed under multiphoton microscopy (Fig. 2a–c). The subcellular localization of TPAs in living MCF7 cells was exclusively cytoplasmic at early times of observation. Two-photon excitation using NIR light triggered cytoplasm→nucleus translocations of both TP3Bzim and TP2Py compounds (Fig. 2a,b, respectively). This effect parallels, at least qualitatively, the one previously observed using visible light irradiation at 458 nm^20^. Two-photon excitation led to re-localization of TPAs into the nucleus, after 6 min of illumination for TP3Bzim (Supplementary Table S1) and 10 min for TP2Py (Supplementary Table S2), corresponding to light doses of 450 and 750 J.cm^−2^ for TP3Bzim and TP2Py, respectively. Blebbing phenomenon as well as cell shrinkage, morphological hallmarks of cell apoptosis, were consistently observed in the transmission mode. Note that plasma membrane blebs were also occasionally directly observed in fluorescence images as shown in Fig. 2c. Based on several criteria such as the beginning of nuclear translocation and the detection of blebs, the effects of TP2Py and TP3Bzim were found to be optimal using λ_exc_ = 860 and 760 nm, respectively (Supplementary Tables S1 and S2), consistent with their respective maximum two-photon absorption wavelengths (Fig. 1a). The higher overall efficiency of TP3Bzim over TP2Py is most likely explained by its higher two-photon absorption cross section (Fig. 1a). Therefore, two-photon excitation induces a fast re-localization process of TPAs and concomitant cell death as previously found using one-photon excitation^20^.

### Insight into the initial localization of TPAs in the cytoplasm

**Figure 3:**
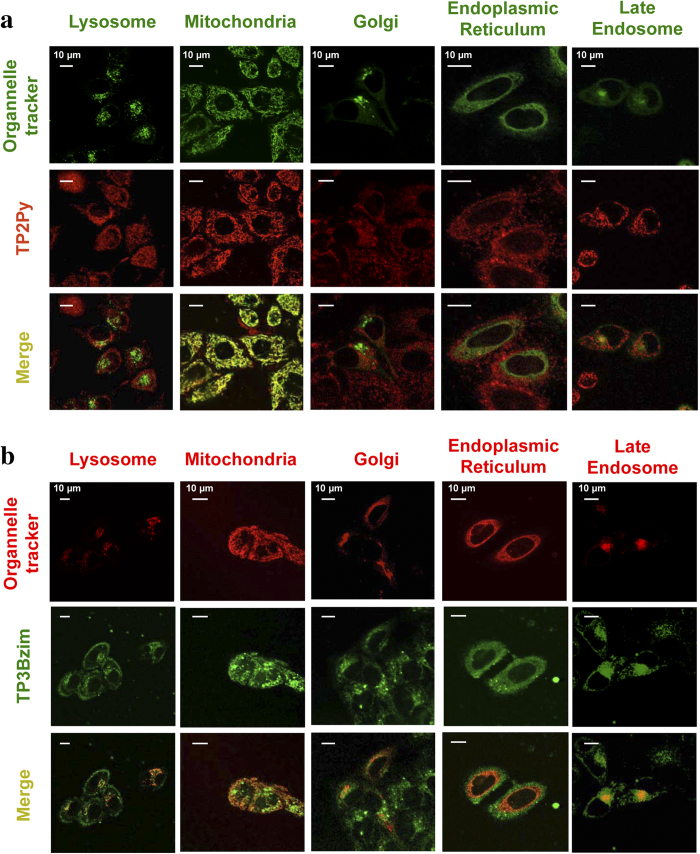
Subcellular localization of TPAs before light-induced nuclear translocation. Colocalization experiments were performed using living HeLa cells, treated with 2 μM TP2Py (**a**) or TP3Bzim (**b**) and further observed using confocal microscopy. Due to its well-marked emission in the red region of the spectrum, the subcellular localization of TP2Py was studied using green trackers of lysosomes or mitochondria, whereas red trackers were used for TP3Bzim. Lysosome and mitochondria staining were performed using LysoTracker^®^ Green DND-26 and MitoTracker^®^ Green FM, respectively, in (panel **a**) (λ_exc_ = 488 nm/emission: 500–550 nm), or LysoTracker^®^ Red DND-99 (λ_exc_ = 543 nm/emission: 600–700 nm) and MitoTracker^®^ Red FM (λ_exc_ = 633 nm/emission: 650–700 nm), respectively, in (panel **b)**. For both TPAs, colocalization experiments with the Golgi apparatus, late endosomes or the endoplasmic reticulum (ER) were performed using HeLa cells expressing the Cerulean protein (blue emission) fused either to the targeting-Golgi Rab6 protein, the targeting-late endosome Rab7 protein or a targeting-ER peptide, respectively (λ_ex_ = 458 nm/emission: 465–510 nm). TPA channel: λ_exc_ = 458 nm/emission slit settings: 560–720 and 530–690 nm for TP2Py and TP3Bzim, respectively. Top: imaging channel of the organelle tracker; middle: imaging channel of TPA; bottom: corresponding merged images. Yellow to orange areas indicate colocalization of TPA/organelle.

Before nuclear translocation upon light illumination, TPAs were initially located in the cytoplasm. A previous analysis of emission spectra in the cellular context suggested that TPAs were not freely-diffusing compounds in the cytoplasm but most likely were sequestered in organelles and, accordingly, we found that at least mitochondria were targets for TP2Py and TP3Bzim^20^. A more systematic characterization of the subcellular localization of TPAs was performed in the present study using a wide range of organelle trackers. Colocalization experiments were then carried out using living cells only, since fixed cells displayed an exclusive nuclear localization of TPAs. For lysosome or mitochondria staining, we used standard commercial LysoTracker or MitoTracker dyes, respectively (green or red, depending on the TPA compound studied) whereas we used three original fusion contructs for colocalization experiments with the endoplasmic reticulum (ER), the Golgi apparatus and late endosomes: the fluorescent Cerulean protein was expressed as a fusion to a ER-peptide targeting sequence or as a fusion to small GTPase Rab6/7 proteins, associated with the Golgi apparatus or the late endosomes, respectively (see Methods for more details). As shown in Fig. 3a, TP2Py strongly colocalized with mitochondria. TP3Bzim also colocalized with mitochondria, however to a lesser extent than TP2Py (Fig. 3b). In the case of TB3Bzim, most of the colocalization signal originated from late endosomes whereas only a weak localization of TP2Py was detected within late endosomes. TP2Py/TP3Bzim did not colocalize with the ER, lysosomes or the Golgi apparatus.

### Evidence for an apoptosis-mediated mechanism of cell death induced by TPAs and differential effects of TP2Py and TP3Bzim

**Figure 4:**
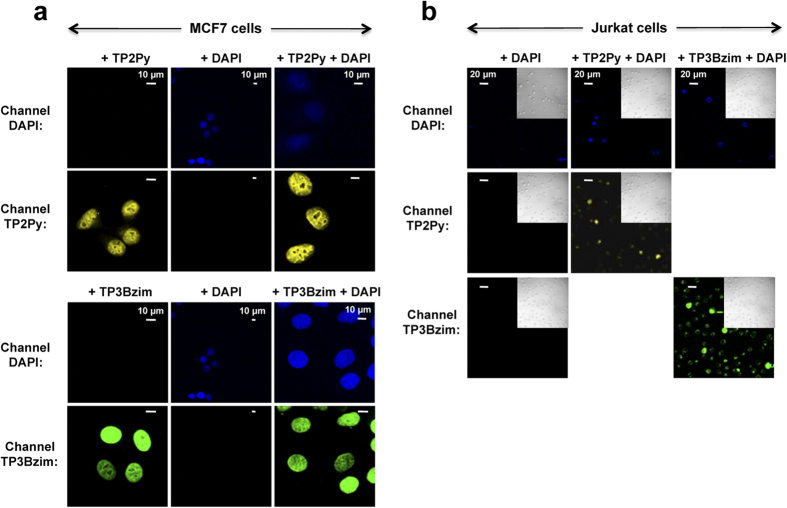
Simultaneous labelling of nuclei by TPAs and DAPI. (**a**) MCF7 cells. Before observation by confocal microscopy, MCF7 cells were fixed with 4% paraformaldehyde and treated with TPA alone (2 μM; left), DAPI alone (200 nM; middle) or a combination of both (2 μM TPA + 200 nM DAPI; right). As already shown in Fig. 1b, TPAs were localized in nuclei under fixation conditions (top: TP2Py; bottom: TP3Bzim). TPA-staining of the nucleus did not prevent DAPI-staining of the nucleus when MCF7 cells were simultaneously treated with TPA and DAPI (right column). (**b**) Jurkat cells. After treatment with DAPI alone (200 nM; left) or DAPI+TPA (200 nM DAPI; 2 μM TPA: middle and right columns for TP2Py and TB3Bzim, respectively), living Jurkat cells were subjected to illumination for 1h by using a Mercury lamp (130 W; 380–600 nm + excitation filter centered at 452 nm (±45 nm); irradiance, 17 mW.cm^−2^) and were further incubated for 4h in the dark before observation by confocal microscopy. Insets: DIC transmission images. No or very weak DAPI-staining of Jurkat nuclei was observed in the absence of any TPA, as expected for living cells (left column). By contrast, some of TPA-treated and illuminated cells displayed DAPI-staining of nuclei (concomitant with nuclear translocation of TPAs), demonstrating that nuclear staining of Jurkat cells by DAPI and TPA is not mutually exclusive under this experimental condition (equivalent to the condition used in flow cytometric analysis shown in Figs 5 & S1). This population of DAPI-stained cells corresponds to necrotic/late apoptotic cells (see Figs 5 & S1). Excitation and emission slit settings: channel TP2Py (λ_exc_ = 458 nm; λ_em_ = 560–720 nm); channel TP3Bzim (λ_exc_ = 458 nm; λ_em_ = 530–690 nm); channel DAPI (λ_exc_ = 740 nm; λ_em_ = 406–506 nm). Incubation times were 2 h and 10 min for TPAs and DAPI, respectively.

The colocalization experiments suggest a relationship between the photocytotoxic effects of TPAs and their common initial mitochondrial localization. Accordingly, we previously shown that photoactivation of TPAs led to the cleavage of the nuclear DNA repair enzyme PARP-1 (poly-ADP ribose polymerase), a target of caspases 3/7, suggesting a cell death mechanism which mainly relies on the caspase-dependent apoptotic pathway^20^. However, the difference in terms of subcellular distributions of TPAs (mitochondria vs late endosomes), as shown above, could be suggestive of divergent mechanisms. To gain deeper insight into the origin and the mechanism of cell death (apoptosis vs necrosis), we measured the effects of irradiated TPAs by the Annexin V assay which probes the externalization of phosphatidylserine at the plasma membrane level^21^. As both apoptotic and necrotic (or late apoptotic) cells lead to Annexin V staining, 4′,6′-diamino-2-phenylindole (DAPI) staining of the nucleus was used simultaneously to distinguish between these two cell subpopulations. DAPI does not permeate cells with intact membranes when used at low concentration (<5μM) and thus allows identification of necrotic/late apoptotic cells only. Taking into account that TPAs are also DNA binders that stain the nucleus during the cell death process, we first demonstrated that nuclear staining by DAPI and TPAs was not mutually exclusive (Fig. 4).

**Figure 5:**
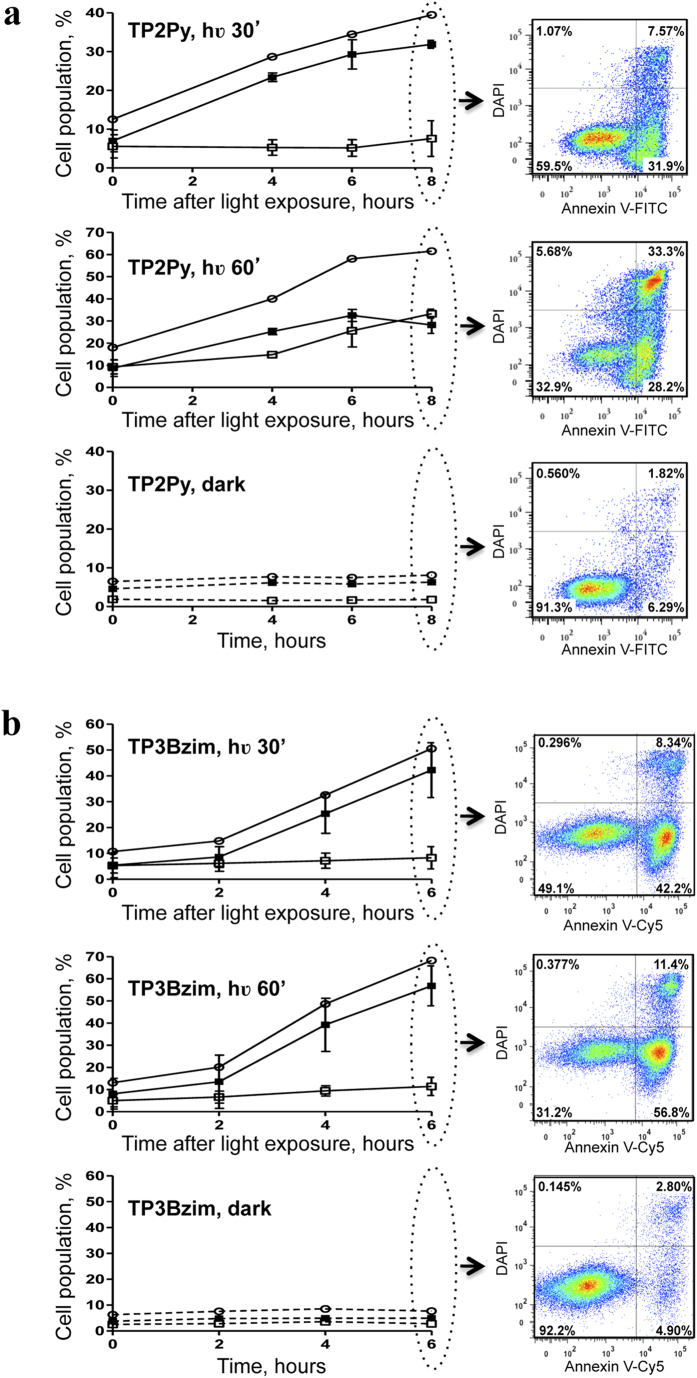
TPAs induce cell apoptosis upon light illumination as revealed by Annexin V/DAPI staining. Living Jurkat cells were treated with 2 μM TP2Py (**a**) or TP3Bzim (**b**) (as indicated in Methods) and subjected to light illumination for 30 (top) or 60 min (middle). The source of excitation was a Mercury lamp (130 W; 380–600 nm) with an excitation filter centered at 452 nm (±45 nm) (irradiance, 17 mW.cm^−2^). The cells were then further incubated for various times in the dark at 37 °C before Annexin V/DAPI treatment and flow cytometric analyses. Cell populations corresponding to apoptotic and necrotic (or late apoptotic) cells were plotted as a function of time after light exposure. Bottom: control population of TPA-treated cells without light exposure. Black squares: apoptotic cells (Annexin V^+^, DAPI^−^); white squares: necrotic/late apoptotic cells (Annexin V^+^, DAPI^+^); white circles: total number of dead cells (Annexin V^+^, DAPI^+/−^). Error bars indicate S.D. (*n* = 3). Right: examples of 2-dimensional dot plots for t = 8h (TP2Py) or 6h (TP3Bzim) (the complete study is shown in Supplementary Fig. 1). All the cells selected for the analysis of Annexin V/DAPI staining were positive for TPA fluorescence.

Non-adherent Jurkat cells were then treated with TPAs, light-exposed and further incubated for various times in the dark before Annexin V/DAPI treatment and flow cytometric analysis (Fig. 5; see also Supplementary Fig. 1 for complete 2-D dot plots). The population of dead cells (Annexin V^+^/DAPI^+/−^) continuously increased after initial light exposure and the overall time course of cell death was light dose-dependent. Again, TP3Bzim led to faster kinetics of cell death than TP2Py, in agreement with previous results on PARP-1 cleavage^20^. In the absence of TPA or in the presence of TPA without light exposure, the population of dead cells was low and constant over time with the percentage of each subpopulation (Annexin V^+^/DAPI^+^and Annexin V^+^/DAPI^−^) remaining unchanged over time. Under the condition of TP2Py treatment and after 30 min of light illumination, the dead cell population was mainly explained by apoptotic cells (Annexin V^+^/DAPI^−^). The other subpopulation (Annexin V^+^/DAPI^+^) remained low and constant up to 6h post-illumination. A small but significant increase of this subpopulation occurred at 8h post-illumination, suggesting that this subpopulation corresponds to necrotic/late apoptotic cells. Using higher light dose conditions (60 min of light illumination), the appearance of the Annexin V^+^/DAPI^+^subpopulation was significantly faster. However, the appearance of Annexin V^+^/DAPI^−^ cells (apoptotic) consistently and chronologically preceded the appearance of Annexin V^+^/DAPI^+^cells (necrotic/late apoptotic). At 8h post-illumination, the latter subpopulation was more represented than the former. TP3Bzim behave similarly to TP2Py under low illumination conditions (30 min). However, the appearance of the Annexin V^+^/DAPI^+^subpopulation was significantly delayed with TP3Bzim compared to TP2Py under high illumination conditions (60 min), despite the overall faster kinetics of cell death, suggesting that TP2Py and TP3Bzim share a common apoptosis pathway but TP2Py also induces a proper necrosis process. Taken together, our results suggest that both TPAs primarily induce apoptosis under light illumination. Notably, our data also show a differential effect of TP2Py/TP3Bzim on the necrotic/late apoptotic subpopulation that may be due to their different subcellular localization profiles.

### Participation of ROS in TPA-mediated apoptosis upon two-photon photoactivation

**Figure 6:**
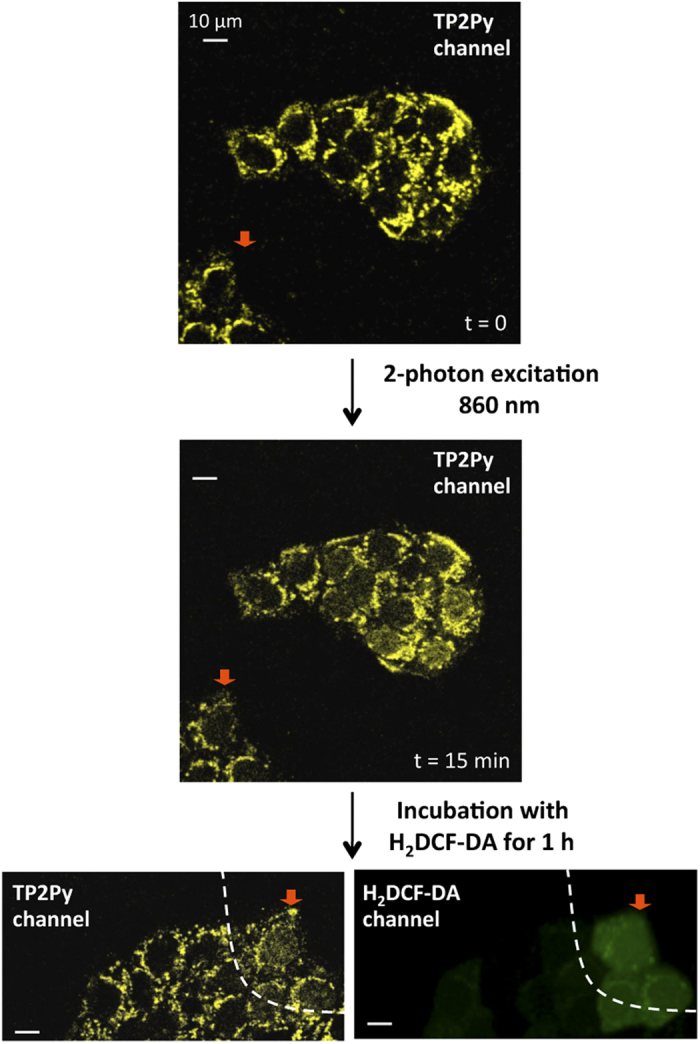
Photoactivation of TPAs promotes ROS production: fluorescence imaging of ROS production upon two-photon excitation of TP2Py. Living MCF7 cells were pre-incubated with 2 μM TP2Py for 2h in the dark at 37 °C and further exposed to two-photon illumination for 15 min (λ_exc_ = 860 nm; irradiance, 1.25 W.cm^−2^). Two-photon fluorescence imaging of TP2Py is presented before (top) or after (middle) 15-min illumination. The cells were then treated with 10 μM H_2_DCF-DA for 1h and the excitation area for imaging was changed by moving the sample (the cell indicated by the red arrow serves as a landmark). Bottom: two-photon and confocal fluorescence imaging of TP2Py (left) and DCF (right), respectively. Excitation wavelengths were 860 nm and 488 nm for TP2Py (emission slit: 560–720 nm) and DCF (emission slit: 500–530 nm), respectively. The fluorescence signal of DCF accounting for ROS production was only detected in cells initially subjected to the 15-min period of light illumination (this area is delineated by a dashed line).

We next addressed the question of whether the two-photon excitation process of TPAs in the cell context leads to a significant ROS production in a similar manner to that observed using visible light irradiation^20^. We used the ROS detector 2′,7′-dichlorodihydrofluorescein diacetate (H_2_DCF-DA) which is converted into the highly fluorescent form DCF in the presence of ROS and directly measured the effect of the concerted action of TPA and light irradiation on ROS production in fluorescence microscopy imaging. As shown in Fig. 6, two-photon (λ_exc_ = 860 nm) irradiation of living MCF7 cells pre-treated with TP2Py led to a specific fluorescence enhancement of DCF in the irradiated area, indicating that photoactivation of TPAs triggered a burst of intracellular concentration of ROS upon two-photon excitation. To note, TP2Py nuclear translocation was systematically associated with ROS production in selected areas (only cells characterized by ROS production also display fluorescence emission of TP2Py at the nucleus level) (Fig. 6). Together, these results indicate that the combination of TP2Py and two-photon irradiation accounts for a significant production of ROS in the cell context, consistent with the initial mitochondrial localization.

## Discussion

Photo-induced cell death in the presence of TPAs as well as the concomitant nuclear translocation of TPAs were found to be efficient upon two-photon excitation with optimal excitation wavelengths of 760 and 860 nm for TP3Bzim and TP2Py, respectively. TPA concentrations used for triggering and simultaneously imaging the overall process (typically in the low micromolar range) are much below the intrinsic toxic concentrations, *i.e.* dark or daylight toxicities (>30 μM)^20^, explaining why ROS production and nuclear translocation of TPAs specifically occur in selected irradiated areas.

Although two- or three-branch TPAs remain localized in the cytoplasm of living cells without affecting cell morphology until photoactivation and behave similarly upon light excitation, we showed here that the cell distribution of the two compounds before activation was not strictly identical; TP2Py fully localized in mitochondria while TP3Bzim localized in both mitochondria and late endosomes, supporting two different modes of TPA uptake: (i) active/passive transport through the plasma membrane and (ii) endocytosis. Taking into account similar intracellular fates and biological effects of TP2Py/TP3Bzim upon light illumination, it is unlikely that TP3Bzim localization in late endosomes plays a primary (direct) role in photo-induced apoptosis, but that the common mitochondrial localization of both TPAs is most likely responsible for apoptosis. However, late endosome-localized TP3Bzim could play an indirect role by being available in the cytosol after illumination, by analogy with the photochemical internalization (PCI) technology^22^. We have previously shown that a neutral TPA derivative (TP3^N^Bzim) has no specific subcellular localization in living cells in contrast to that observed with TP2Py or TP3Bzim^20^. Furthermore, TP3^N^Bzim was unable to trigger cell apoptosis under light irradiation. TP2Py/TP3Bzim belong to a class of delocalized cations, a specific feature found in a number of already identified small mitochondria-targeting compounds^23^,^24^,^25^,^26^, some of them are efficient two-photon fluorescent probes for imaging mitochondria^26^,^27^,^28^,^29^. They primarily induce apoptosis upon light illumination, in accordance with their initial mitochondrial localization. This is also consistent with the fact that most of the PDT compounds targeting mitochondria (or ER) induce apoptosis whereas other PDT compounds directly targeting the plasma membrane (or lysosomes) preferentially lead to necrosis^30^. However, TP2Py and TP3Bzim display distinct temporal responses after initial illumination regarding the balance between apoptosis and late apoptosis/necrosis. Alternatively, the difference in subcellular localization profiles of TP2Py/TP3Bzim could account for the observed delay in the appearance of late apoptotic/necrotic cells in the case of TP3Bzim although the mechanism behind remains unclear. Importantly, this delay cannot be simply explained by differences in spectral properties of TP2Py and TP3Bzim. Indeed, such differences most likely account for the overall faster kinetics of cell death observed with TP3Bzim compared to TP2Py (higher light absorption by TP3Bzim at ≈450 nm in the one-photon set-up and σ^2^_TP3Bzim_ > σ^2^_TP2Py_ in two-photon experiments). The appearance of late apoptotic/necrotic cells which occurs faster with TP2Py, suggests that the time courses of biochemical events leading to cell death intrinsically differ for the two TPA compounds.

Two-photon photoactivation of TPAs led to ROS formation as measured directly by DCF fluorescence in the cell context. Most likely, TPAs account for initial ROS production via type-1 (H_2_O_2_, O_2_^•^, ^•^OH) and/or type-2 (^1^O_2_) photochemical reactions in a manner similar to that of conventional PDT compounds^30^,^31^. Highly fluorescent molecules have generally low singlet oxygen quantum yields. Accordingly, this quantum yield was ≈0.04 for TP3Bzim (measured in DMF). However, the initial and common mitochondrial localization for TP2Py/TP3Bzim suggest that ROS production is amplified at the mitochondrial level, a consequence of mitochondrial permeability transition, to reach toxic concentrations^32^. Accordingly, the mitochondrial localization appears to be preferable for efficient PDT^33^. Moreover, the protection effect of cyclosporine A (a compound that inhibits mitochondrial permeability transition) on the ROS production upon TPA photoactivation, reinforces this idea^20^. Finally, mitochondrial membrane depolarization also accounts for the subcellular re-localization of TPAs (from the cytoplasm to the nucleus) and taking into account their DNA-binding properties, TPAs could lead to DNA damage, but this is probably not the primary cause of rapid cell death as observed in our study, in accordance with many other PDT compounds^34^.

In two-photon experiments, the fluence values (minimally 188–750 J.cm^−2^; Table 1) were found to be significantly higher compared with corresponding values in one-photon experiments (typically in the 15–144 J/cm^2^ range; Table 1). However, these values remain compatible with non-invasive illumination conditions used *in vivo* (animals and patients) and significantly below the photodamage threshold^10^,^35^,^36^,^37^,^38^. Moreover, the laser power as measured at the entrance of the objective in our two-photon experiments was 20 mW, a value considered as standard in non-invasive *in vivo* two-photon studies^39^,^40^,^41^,^42^. In summary, TPAs act simultaneously as pro-apoptotic agents (two-photon PDT) and apoptotic tracers, thereby displaying suitable features as potential theranostic agents. Two-photon PDT is a promising approach that significantly improves the penetration depth of light in tissues and as much may contribute to establish PDT as a realistic alternative to chemotherapy/radiotherapy for cancer treatment. However, the low number of biocompatible organic compounds directly activatable by NIR light is one of the major limitations for the development of therapeutic applications of two-photon PDT. The chemical properties and photocytotoxicity profiles of TPAs, as well as their large two-photon absorption cross-sections represent important features to expand the use of two-photon PDT.

## Methods

### Synthesis and reagents

Synthesis of TP2Py (4,4′-bis[(E)-2-(pyridin-4-yl)vinyl]triphenylamine bis-methiodide) and TP3Bzim (2,2′,2″-((1E,1′E,1″E)-(nitrilotris(benzene-4,1-diyl))tris-(ethene-2,1-diyl))tris(1,3-dimethyl-1H-benzo[d]imidazol-3-ium)iodide) were performed as previously described^18^,^19^. 2′,7′-dichlorodihydrofluorescein diacetate (H_2_DCF-DA) and 4′,6′-diamidino-2-phenylindole (DAPI) were purchased from Sigma-Aldrich and Invitrogen, respectively.

### Spectroscopic characterization and instrument set-up for fluorescence imaging

Absorption spectra of TPAs in Tris buffer (10 mM Tris-HCl, pH 7.2, 100 mM KCl) were carried out with a Uvikon spectrophotometer. Two-photon excitation and emission spectra of TPAs in Tris buffer were recorded using a home-built set-up^43^. Briefly, a 80-MHz mode-locked Mai-Tai^®^ Ti:Sapphire tunable laser (690–1040 nm, 100 fs laser pulse; Spectra Physics, Mountain View, California) was focused onto the sample (80 μl) placed in a quartz micro cell. The two-photon fluorescence was collected at 90 degrees and filtered by a Semrock FF01-842/SP filter to reject the residual excitation light. The fluorescence signal was focused into an optical fiber connected to a QE65000 spectrometer (Ocean Optics). The TPA concentration was 5 μM in the presence or absence of 10 μM 21-mer double-stranded DNA (U5A/B); U5A (5′-cct gct agg gat ttt cct gcc-3′) and its complementary B strand were annealed according to^44^,^45^,^46^.

Confocal images (colocalization experiments and imaging of H_2_DCF-DA) were obtained using a SP2 confocal microscope (Leica MicroSystem) equipped with an oil immersion ×63 objective (numerical aperture, 1.32) and an incubation chamber (37 °C, CO_2_ 5%). A continuous laser line (458 nm) was used for excitation of TPAs in the confocal mode (emission slit settings are specified in figure legends). A similar set-up was used for two-photon images except that the excitation source was a 80-MHz mode-locked Mai-Tai^®^ Ti:Sapphire tunable laser (720–920 nm, 100 fs laser pulse; Spectra Physics, Mountain View, California) tuned to 760 or 860 nm for TP3Bzim and TP2Py, respectively. The irradiances were calculated by measuring the power at the exit of the objective using a Vega power meter (Ophir). The power measurement was performed in the x-y scan mode (image size, 512 × 512 pixels; field of view, 140 × 140 μm; scanning frequency, 800 Hz). z-stack in two-photon microscopy corresponded to 10 slices (512 × 512 pixels; z-step, 1 μm). The time scale accounting for the effective illumination/excitation time in two-photon imaging experiments was adjusted according to the z-stack to allow comparison with previously confocal data^20^ since the multiphoton excitation process intrinsically provides optical sectioning (discrimination occurs at excitation) whereas the z-resolution in confocal microscopy is achieved by a pinhole in the emission path that eliminates out-of-focus light (no discrimination at excitation along the z-axis).

### Constructions of plasmid and lentiviral shuttle vectors for colocalization experiments

#### Construction of the pShooter-ER-Cerulean plasmid

Amplification of the cDNA encoding the Cerulean protein was performed from the LeGO-Cer2 plasmid^47^ by PCR using two primers, PR1 (5′-ggg tcg acg tga gca agg gc-3′) and PR2 (5′-gcg gcc gcc ttg tac agc tcg-3′), introducing two restriction sites, Sal1 after the atg sequence and Not1 before the stop codon, respectively. The PCR-product was subcloned into the pJET2.1 plasmid (CloneJET PCR cloning kit; Thermo Scientific). After digestion of the resulting plasmid (pJET-Cer-Sal/Not) by Sal1/Not1, the 714-bp fragment containing the *cer* gene was ligated (Quick ligase; Biolabs) into the pShooter-ER plasmid digested by Sal1/Not1 to yield the pShooter-ER-Cerulean plasmid.

#### Construction of the lentiviral shuttle vector expressing the Cerulean-rab6 fusion gene

Firstly, amplification of the cDNA encoding the eGFP-Rab6 fusion protein was performed from the peGFPC2Rab6 plasmid (kindly provided by M-H. Kryske, LBPA, ENS Cachan, France) by PCR using two primers, PR3 (5′-gtc gac atg gtg agc aag g-3′) and PR4 (5′-ggt acc tta gca gga aca gc-3′), introducing two restriction sites, Sal1 before the atg sequence and Kpn1 after the stop codon, respectively. The PCR-product was subcloned into the pJET2.1 plasmid. After digestion of the resulting plasmid (pJET-eGFP-Rab6) by Sal1/Kpn1, the 1383-bp fragment containing the *eGFP-rab6* gene was ligated into a lentiviral shuttle vector (pHR′ CMV-MCS) digested by Sal1/Kpn1 to yield the pHR′-eGFP-Rab6 vector. Secondly, the *cer* gene was substituted for the *eGFP* gene. Briefly, the cDNA encoding the Cerulean protein was amplified from the LeGO-Cer2 plasmid by PCR using two primers, PR3 and PR5 (5′-gaa ttc cct tgt aca gct cg-3′), introducing two restriction sites, Sal1 before the atg sequence and EcoR1 before the stop codon, respectively. A cytosine base was introduced between the *cer* gene and the EcoR1 restriction site to preserve the reading frame after the *eGFP* −>*cer* substitution. The PCR-product was subcloned into the pJET2.1 plasmid. After digestion of the resulting plasmid (pJET-Cer-Sal/Eco) by Sal1/EcoR1, the 718-bp fragment containing the *cer* gene was ligated into the pHR′-eGFP-Rab6 vector digested by Sal1/EcoR1 to yield the pHR′-Cer-Rab6 vector (an EcoR1 restriction site is present between the *eGFP* and *rab6* genes).

#### Construction of the lentiviral shuttle vector expressing the Cerulean-rab7 fusion gene

Amplification of the cDNA encoding the Rab7 protein was performed from the DsRed-Rab7 plasmid (Addgene plasmid 12661) by PCR using two primers, PR6 (5′-gaa ttc gta cct cta gga ag-3′) and PR7 (5′-ggt acc tca gca act gca g-3′), introducing two restriction sites, EcoR1 after the atg sequence and Kpn1 after the stop codon, respectively. Two bases (gt) were introduced between the EcoR1 restriction site and the *rab7* gene to preserve the reading frame after the *rab6* −>*rab7* substitution. The PCR-product was subcloned into the pJET2.1 plasmid. After digestion of the resulting plasmid (pJET-Rab7) by EcoR1/Kpn1, the 623-bp fragment containing the *rab7* gene was ligated into the pHR′-Cer-Rab6 vector digested by EcoR1/Kpn1 to yield the pHR′-Cer-Rab7 vector.

### Colocalization experiments

All colocalization experiments were performed on living HeLa cells using confocal microscopy (excitation/emission settings are indicated in the corresponding figure legend).

#### Colocalization experiments with lysosome and mitochondria

HeLa cells were pre-treated with TPA (2 μM) for 2 hours. The cells were then washed with PBS buffer (phosphate-buffered saline, pH 7.5, Gibco^®^) and further incubated with either 50 nM LysoTracker^®^ (Invitrogen) for 90 min or 20 nM MitoTracker^®^ (Invitrogen) for 30 min, for lysosome or mitochondria staining, respectively. For TP2Py (red emission), organelle trackers characterized by green emission were used: LysoTracker^®^ Green DND-26 and MitoTracker^®^ Green FM. For TP3Bzim (green emission), organelle trackers characterized by red emission were used: LysoTracker^®^ Red DND-99 and MitoTracker^®^ Red FM.

#### Colocalization experiments with the endoplasmic reticulum (ER)

The gene encoding the Cerulean protein (from the LeGO-Cer2 plasmid) was introduced into the pShooter-ER plasmid (Invitrogen) by PCR as mentioned above. Six days after calcium phosphate-mediated transfection of HeLa cells with the resulting pShooter-ER-Cerulean plasmid (allowing expression of the Cerulean as a fusion to a ER-targeting sequence), cells stably expressing the Cerulean protein at the ER level were sorted using a FACSAria cell sorter (Becton-Dickinson) and further incubated with 2 μM of either TPA for 2h before colocalization experiments.

#### Colocalization experiments with the Golgi apparatus and the late endosomes

For labelling the Golgi apparatus or the late endosomes, the Cerulean protein was expressed as a fusion to Rab6 or Rab7 (see above), two small GTPase proteins associated with the Golgi apparatus or the late endosomes, respectively. A lentiviral shuttle vector (pHR′ CMV-MCS, Vectalys) carrying a puromycin resistance gene (under the control of the LTR-5′ promoter) and the *Cerulean-rab6* (or *Cerulean-rab7*) fusion gene (under the control of the CMV promoter) was used for co-transfection of HEK293T cells, together with pMDG (encoding the VSVg envelope protein) and p8.74 (encoding the Gag-Pol proteins). The resulting viruses (obtained by trans-complementation) were used for the infection of HeLa cells in the presence of 5 μg/ml puromycin. The selected cells were then incubated with 2 μM of either TPA for 2h before colocalization experiments.

### Annexin V/DAPI staining

Non-adherent Jurkat cells were plated in 12-well plates (10^6^ cells/well), pre-incubated with 2 μM TP2Py or TP3Bzim for 2h in the dark at 37 °C and subjected to light illumination for either 30 or 60 min at 17 mW.cm^−2^ (Mercury lamp (130 W; 380–600 nm)+ excitation filter centered at 452 nm (±45 nm); total illuminated surface: 3.8 cm^2^ (equiv. 1 well)). After light exposure, the cells were either immediately (t = 0) treated for Annexin V/DAPI staining or further incubated for various times (up to t = 8h) in the dark at 37 °C before Annexin V/DAPI treatment (Annexin V-FITC or Annexin V-Cy5 were used for experiments in the presence of TP2Py or TP3Bzim, respectively). Briefly, the cells were washed in ice-cold PBS 1× (Gibco^®^) and stained according to the manufacturer’s instructions (BD Biosciences); the cell pellet was re-suspended in 100 μl Annexin V-HEPES solution (Annexin V diluted in the binding buffer: 10 mM HEPES-NaOH, pH 7.4, 140 mM NaCl, 2.5 mM CaCl_2_) and incubated for 20 min in the dark at room temperature. After addition of 400 μl of binding buffer containing DAPI (200 nM), the cells were further incubated for 10 min. Cells were then counted by flow cytometry (FACSCantoII flow cytometer; Becton-Dickinson). The laser/detection channels for fluorescence were 405-B for DAPI (exc: 405 nm, em: 450 ± 25 nm), 488-E for Annexin V-FITC and TP3Bzim (exc: 488 nm, em: 530 ± 15 nm), 488-D for TP2Py (exc: 488 nm, em: 585 ± 20 nm) and 633-C for Annexin V-Cy5 (exc: 633 nm, em: 660 ± 10 nm).

### Fluorescence detection of ROS generation

Direct ROS detection in fluorescence imaging experiments was performed with H_2_DCF-DA as explained in the corresponding figure legend. Two-photon excitation and fluorescence imaging of TP2Py was performed using a tunable pulsed laser source (λ_exc_ = 860 nm; emission slit setting: 560–720 nm) whereas a continuous laser line (488 nm) was used for excitation and imaging of DCF (emission slit setting: 500–530 nm).

## Additional Information

**How to cite this article**: Chennoufi, R. *et al.* Mitochondria-targeted Triphenylamine Derivatives Activatable by Two-Photon Excitation for Triggering and Imaging Cell Apoptosis. *Sci. Rep.* **6**, 21458; doi: 10.1038/srep21458 (2016).

## Supplementary Material

Supplementary Information

## Figures and Tables

**Table 1:**
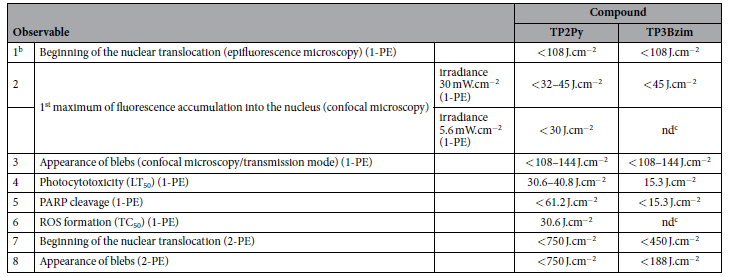
Indicative values of fluences^a^ characterizing TPA compounds based on morphological and biochemical criteria.

1-PE: one-photon excitation; 2-PE: two-photon excitation.

^a^Fluences were calculated for a TPA concentration of 2 μM. The symbol “<” means that fluence was measured under continuous illumination and then corresponds to an upper limit value.

^b^**1–6**: from ref. 20; **7–8**: Supplementary Tables S1 and S2. ^c^nd: not determined.

## References

[b1] Dolmans D. E., Fukumura D. & Jain R. K. Photodynamic therapy for cancer. Nat. Rev. Cancer 3, 380–387 (2003). 12724736 10.1038/nrc1071

[b2] Lim C. K. *et al.* Nanophotosensitizers toward advanced photodynamic therapy of Cancer. Cancer Lett. 334, 176–187 (2013). 23017942 10.1016/j.canlet.2012.09.012

[b3] Ferrario A., Rucker N., Wong S., Luna M. & Gomer C. J. Survivin, a member of the inhibitor of apoptosis family, is induced by photodynamic therapy and is a target for improving treatment response. Cancer Res. 67, 4989–4995 (2007). 17510430 10.1158/0008-5472.CAN-06-4785

[b4] Szokalska A. *et al.* Proteasome inhibition potentiates antitumor effects of photodynamic therapy in mice through induction of endoplasmic reticulum stress and unfolded protein response. Cancer Res. 69, 4235–4243 (2009). 19435917 10.1158/0008-5472.CAN-08-3439 PMC2785802

[b5] Anand S., Wilson C., Hasan T. & Maytin E. V. Vitamin D3 enhances the apoptotic response of epithelial tumors to Aminolevulinate-based photodynamic therapy. Cancer Res. 71, 6040–6050 (2011). 21807844 10.1158/0008-5472.CAN-11-0805 PMC3360482

[b6] Nakajima T., Sano K., Mitsunaga M., Choyke P. L. & Kobayashi H. Real-time monitoring of *in vivo* acute necrotic cancer cell death induced by near infrared photoimmunotherapy using fluorescence lifetime imaging. Cancer Res. 72, 4622–4628 (2012). 22800710 10.1158/0008-5472.CAN-12-1298 PMC3445723

[b7] Starkey J. R. *et al.* New two-photon activated photodynamic therapy sensitizers induce xenograft tumor regressions after near-IR laser treatment through the body of the host mouse. Clin. Cancer Res. 14, 6564–6573 (2008). 18927297 10.1158/1078-0432.CCR-07-4162

[b8] Idris N. M. *et al.* *In vivo* photodynamic therapy using upconversion nanoparticles as remote-controlled nanotransducers. Nat. Med. 18, 1580–1585 (2012). 22983397 10.1038/nm.2933

[b9] Kachynski A. V. *et al.* Photodynamic therapy by *in situ* nonlinear photon conversion. Nature Photon. 8, 455–461 (2014).

[b10] Karotki A., Khurana M., Lepock J. R. & Wilson B. C. Simultaneous two-photon excitation of photofrin in relation to photodynamic therapy. Photochem. Photobiol. 82, 443–452 (2006). 16613497 10.1562/2005-08-24-RA-657

[b11] Khurana M. *et al.* Quantitative *in vitro* demonstration of two-photon photodynamic therapy using Photofrin^®^ and Visudyne^®^. Photochem. Photobiol. 83, 1441–1448 (2007). 18028219 10.1111/j.1751-1097.2007.00185.x

[b12] Gao D., Agayan R. R., Xu H., Philbert M. A. & Kopelman R. Nanoparticles for two-photon photodynamic therapy in living cells. Nano Lett. 6, 2383–2386 (2006). 17090062 10.1021/nl0617179 PMC2577608

[b13] Dahlstedt E. *et al.* One- and two-photon activated phototoxicity of conjugated porphyrin dimers with high two-photon absorption cross sections. Org. Biomol. Chem. 7, 897–904 (2009). 19225672 10.1039/b814792b

[b14] Kim S., Ohulchanskyy T. Y., Pudavar H. E., Pandey R. K. & Prasad P. N. Organically modified silica nanoparticles co-encapsulating photosensitizing drug and aggregation-enhanced two-photon absorbing fluorescent dye aggregates for two-photon photodynamic therapy. J. Am. Chem. Soc. 129, 2669–2675 (2007). 17288423 10.1021/ja0680257 PMC2556058

[b15] Zhao T. *et al.* Gold nanorod enhanced two-photon excitation fluorescence of photosensitizers for two-photon imaging and photodynamic therapy. ACS Appl. Mater. Interfaces 6, 2700–2708 (2014). 24483257 10.1021/am405214w

[b16] Gallavardin T. *et al.* An improved singlet oxygen sensitizer with two-photon absorption and emission in the biological transparency window as a result of ground state symmetry-breaking. Chem. Commun. 48, 1689–1691 (2012). 10.1039/c2cc15904j 22182988

[b17] Allain C. *et al.* Vinyl-pyridinium triphenylamines: novel far-red emitters with high photostability and two-photon absorption properties for staining DNA. Chembiochem. 8, 424–433 (2007). 17279593 10.1002/cbic.200600483

[b18] Dumat B. *et al.* DNA switches on the two-photon efficiency of an ultrabright triphenylamine fluorescent probe specific of AT regions. J. Am. Chem. Soc. 135, 12697–12706 (2013). 23914799 10.1021/ja404422z

[b19] Dumat B. *et al.* Vinyl-triphenylamine dyes, a new family of switchable fluorescent probes for targeted two-photon cellular imaging: from DNA to protein labeling. Org. Biomol. Chem. 10, 6054–6061 (2012). 22614341 10.1039/c2ob25515d

[b20] Chennoufi R. *et al.* Differential behaviour of cationic triphenylamine derivatives in fixed and living cells. Chem. Commun. 51, 14881–14884 (2015). 10.1039/c5cc05970d 26303028

[b21] Kuznetsov G. *et al.* Induction of morphological and biochemical apoptosis following prolonged mitotic blockage by halichondrin B macrocyclic ketone analog E7389. Cancer Res. 64, 5760–5766 (2004). 15313917 10.1158/0008-5472.CAN-04-1169

[b22] Berg K. *et al.* Photochemical internalization: a new tool for gene and oligonucleotide delivery. Top. Curr. Chem. 296, 251–281 (2010). 21504105 10.1007/128_2010_63

[b23] Engelmann F. M. *et al.* Interaction of cationic meso-porphyrins with liposomes, mitochondria and erythrocytes. J. Bioenerg. Biomembr. 39, 175–185 (2007). 17436065 10.1007/s10863-007-9075-0

[b24] Magut P. K. *et al.* Tunable cytotoxicity of rhodamine 6G via anion variations. J. Am. Chem. Soc. 135, 15873–15879 (2013). 24059469 10.1021/ja407164w PMC4197813

[b25] Yousif L. F., Stewart K. L. & Kelley S. O. Targeting mitochondria with organelle-specific compounds: strategies and applications. Chembiochem. 10, 1939–1950 (2009). 19637148 10.1002/cbic.200900185

[b26] Kim H. M. & Cho B. R. Small-molecule two-photon probes for bioimaging applications. Chem. Rev. 115, 5014–5055. 25938620 10.1021/cr5004425

[b27] Bae S.K. *et al.* A ratiometric two-photon fluorescent probe reveals reduction in mitochondrial H2S production in Parkinson’s disease gene knockout astrocytes. J. Am. Chem. Soc. 135, 9915–9923 (2013). 23745510 10.1021/ja404004v

[b28] Liu X. *et al.* A series of carbazole cationic compounds with large two-photon absorption cross sections for imaging mitochondria in living cells with two-photon fluorescence microscopy. J. Fluoresc. 21, 497–506 (2011). 20953825 10.1007/s10895-010-0736-8

[b29] Yang W. *et al.* Two-photon fluorescence probes for imaging of mitochondria and lysosomes. Chem. Commun. 49, 3428–3430 (2013). 10.1039/c3cc41240g 23503659

[b30] Buytaert E., Dewaele M. & Agostinis P. Molecular effectors of multiple cell death pathways initiated by photodynamic therapy. Biochim. Biophys. Acta 1776, 86–107 (2007). 17693025 10.1016/j.bbcan.2007.07.001

[b31] Plaetzer K., Krammer B., Berlanda J., Berr F. & Kiesslich T. Photophysics and photochemistry of photodynamic therapy: fundamental aspects. Lasers Med. Sci. 24, 259–268 (2009). 18247081 10.1007/s10103-008-0539-1

[b32] Circu M. L. & Aw T. Y. Reactive oxygen species, cellular redox systems, and apoptosis. Free Radic. Biol. Med. 48, 749–762 (2010). 20045723 10.1016/j.freeradbiomed.2009.12.022 PMC2823977

[b33] Oliveira C. S., Turchiello R., Kowaltowski A. J., Indig G. L. & Baptista M. S. Major determinants of photoinduced cell death: Subcellular localization versus photosensitization efficiency. Free Radic. Biol. Med. 51, 824–833 (2011). 21664269 10.1016/j.freeradbiomed.2011.05.023

[b34] Robertson C. A., Evans D. H. & Abrahamse H. Photodynamic therapy (PDT): a short review on cellular mechanisms and cancer research applications for PDT. J. Photochem. Photobiol. B 96, 1–8 (2009). 19406659 10.1016/j.jphotobiol.2009.04.001

[b35] Brown S. B., Brown E. A. & Walker I. The present and future role of photodynamic therapy in cancer treatment. Lancet Oncol. 5, 497–508 (2004). 15288239 10.1016/S1470-2045(04)01529-3

[b36] Moore C. M., Pendse D. & Emberton M. Photodynamic therapy for prostate cancer–a review of current status and future promise. Nat. Clin. Pract. Urol. 6, 18–30 (2009). 19132003 10.1038/ncpuro1274

[b37] Moisenovich M. M. *et al.* Novel photosensitizers trigger rapid death of malignant human cells and rodent tumor transplants via lipid photodamage and membrane permeabilization. PLoS One 5, e12717 (2010). 20856679 10.1371/journal.pone.0012717 PMC2939899

[b38] Usuda J. *et al.* Outcome of photodynamic therapy using NPe6 for bronchogenic carcinomas in central airways >1.0 cm in diameter. Clin. Cancer Res. 16, 2198–2204 (2010). 20332318 10.1158/1078-0432.CCR-09-2520

[b39] Sacconi L., Dombeck D. A. & Webb W. W. Overcoming photodamage in second-harmonic generation microscopy: real-time optical recording of neuronal action potentials. Proc. Natl. Acad. Sci. USA 103, 3124–3129 (2006). 16488972 10.1073/pnas.0511338103 PMC1413939

[b40] Kobat D. *et al.* Deep tissue multiphoton microscopy using longer wavelength excitation. Opt. Express 17, 13354–13364 (2009). 19654740 10.1364/oe.17.013354

[b41] Wang B. G., Konig K. & Halbhuber K. J. Two-photon microscopy of deep intravital tissues and its merits in clinical research. J. Microsc. 238, 1–20 (2010). 20384833 10.1111/j.1365-2818.2009.03330.x

[b42] Steven P., Bock F., Huttmann G. & Cursiefen C. Intravital two-photon microscopy of immune cell dynamics in corneal lymphatic vessels. PLoS One 6, e26253 (2011). 22028842 10.1371/journal.pone.0026253 PMC3197633

[b43] Li Y. *et al.* Rational design of a fluorescent NADPH derivative imaging constitutive nitric-oxide synthases upon two-photon excitation. Proc. Natl. Acad. Sci. USA 109, 12526–12531 (2012). 22802674 10.1073/pnas.1205645109 PMC3411954

[b44] Pinskaya M. *et al.* HIV-1 integrase complexes with DNA dissociate in the presence of short oligonucleotides conjugated to acridine. Biochemistry 43, 8735–8743 (2004). 15236582 10.1021/bi049706m

[b45] Delelis O. *et al.* Insight into the integrase-DNA recognition mechanism. A specific DNA-binding mode revealed by an enzymatically labeled integrase. J. Biol. Chem. 283, 27838–27849 (2008). 18697740 10.1074/jbc.M803257200

[b46] Carayon K. *et al.* A cooperative and specific DNA-binding mode of HIV-1 integrase depends on the nature of the metallic cofactor and involves the zinc-containing N-terminal domain. Nucleic Acids Res. 38, 3692–3708 (2010). 20164093 10.1093/nar/gkq087 PMC2887959

[b47] Weber K. *et al.* RGB marking facilitates multicolor clonal cell tracking. Nat. Med. 17, 504–509 (2011). 21441917 10.1038/nm.2338

