# Supplementary material for: Mitochondria-targeted Triphenylamine Derivatives Activatable by Two-Photon Excitation for Triggering and Imaging Cell Apoptosis
Source: Sci Rep. 2016 Mar 7;6:21458. doi: 10.1038/srep21458 (PMC4780088; doi:10.1038/srep21458)
Supplement: Supplementary Information [file srep21458-s1.pdf]

SUPPORTING INFORMATION

Mitochondria-targeted Triphenylamine Derivatives Activatable by Two-Photon Excitation for Triggering and Imaging Cell Apoptosis

Rahima Chennoufi[1], Houcine Bougherara[1], Nathalie Gagey-Eilstein[1,3], Blaise Dumat[2], Etienne Henry[1,4], Frédéric Subra[1], Stéphanie Bury-Moné[1], Florence Mahuteau-Betzer[2], Patrick Tauc[1], Marie-Paule Teulade-Fichou[2*] and Eric Deprez[1*]

[1]Laboratory of Biology and Applied Pharmacology, ENS Cachan, CNRS UMR8113, IDA FR3242, F-94235 Cachan, France

[2]Laboratory of Chemistry, CNRS UMR176, Institut Curie, University of Orsay, Paris-Sud, 91405 Orsay, France

[3]Present address: CNRS UMR8638, University of Paris-Descartes, 75006 Paris, France

[4]Present address: CNRS UMR6197 / UBO / IFREMER, 29280 Plouzane, France

[*]corresponding authors:
E-mail: deprez@lbpa.ens-cachan.fr; mp.teulade-fichou@curie.fr

Table of contents:

- Figure S1a……………………………..………..……………………page 2
- Figure S1b……………………………..………..……………………page 3
- Table S1……..…………………………………………………..……....page 4
- Table S2……..…………………………………………………..……....page 4

a

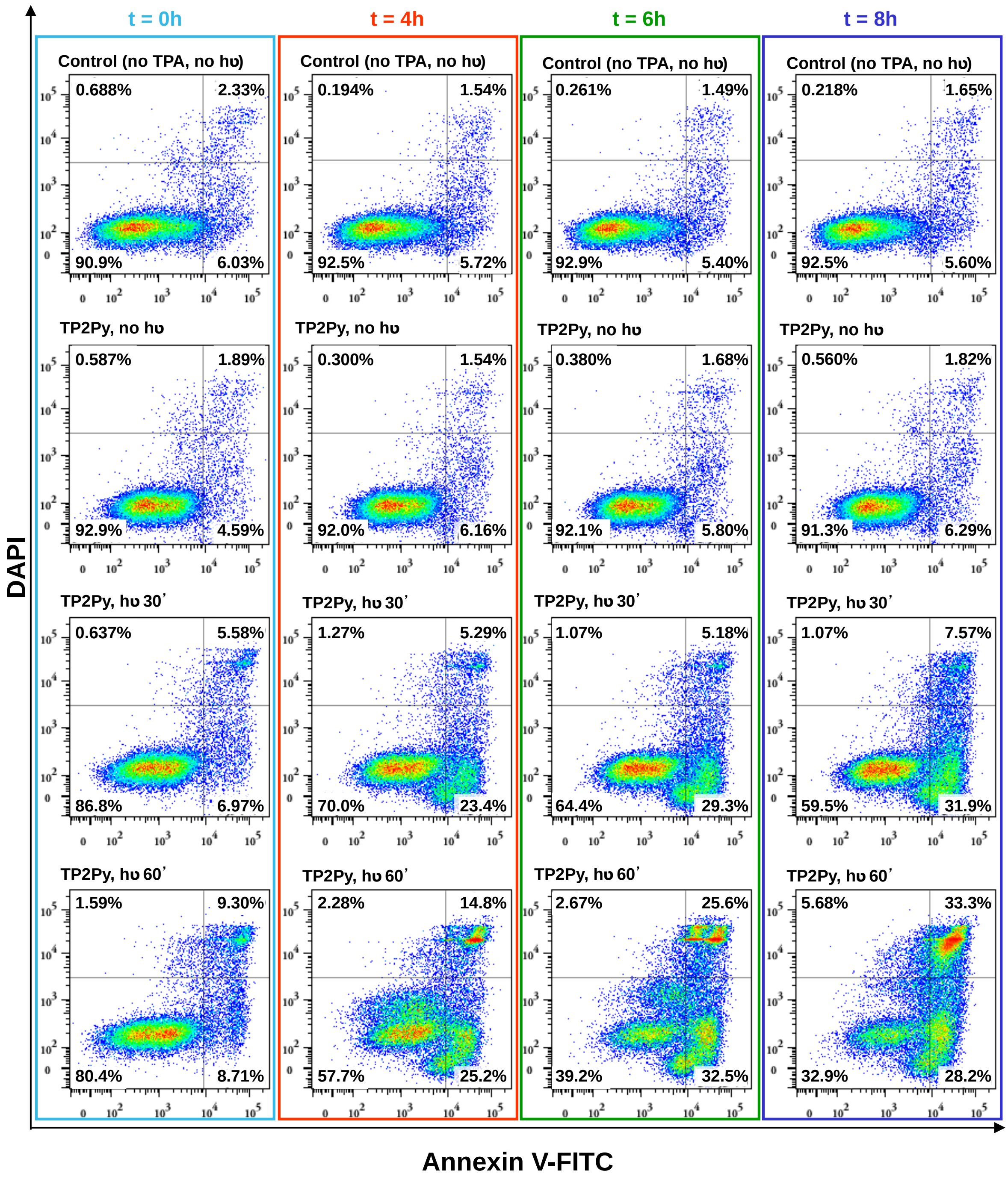
t = 0h
t = 4h
t = 6h
t = 8h
Control (no TPA, no hυ)
0.688%
2.33%
90.9%
6.03%
0.194%
1.54%
92.5%
5.72%
0.261%
1.49%
92.9%
5.40%
0.218%
1.65%
92.5%
5.60%
TP2Py, no hυ
0.587%
1.89%
92.9%
4.59%
0.300%
1.54%
92.0%
6.16%
0.380%
1.68%
92.1%
5.80%
0.560%
1.82%
91.3%
6.29%
TP2Py, hυ 30'
0.637%
5.58%
86.8%
6.97%
1.27%
5.29%
70.0%
23.4%
1.07%
5.18%
64.4%
29.3%
1.07%
7.57%
59.5%
31.9%
TP2Py, hυ 60'
1.59%
9.30%
80.4%
8.71%
2.28%
14.8%
57.7%
25.2%
2.67%
25.6%
39.2%
32.5%
5.68%
33.3%
32.9%
28.2%
DAPI
Annexin V-FITC

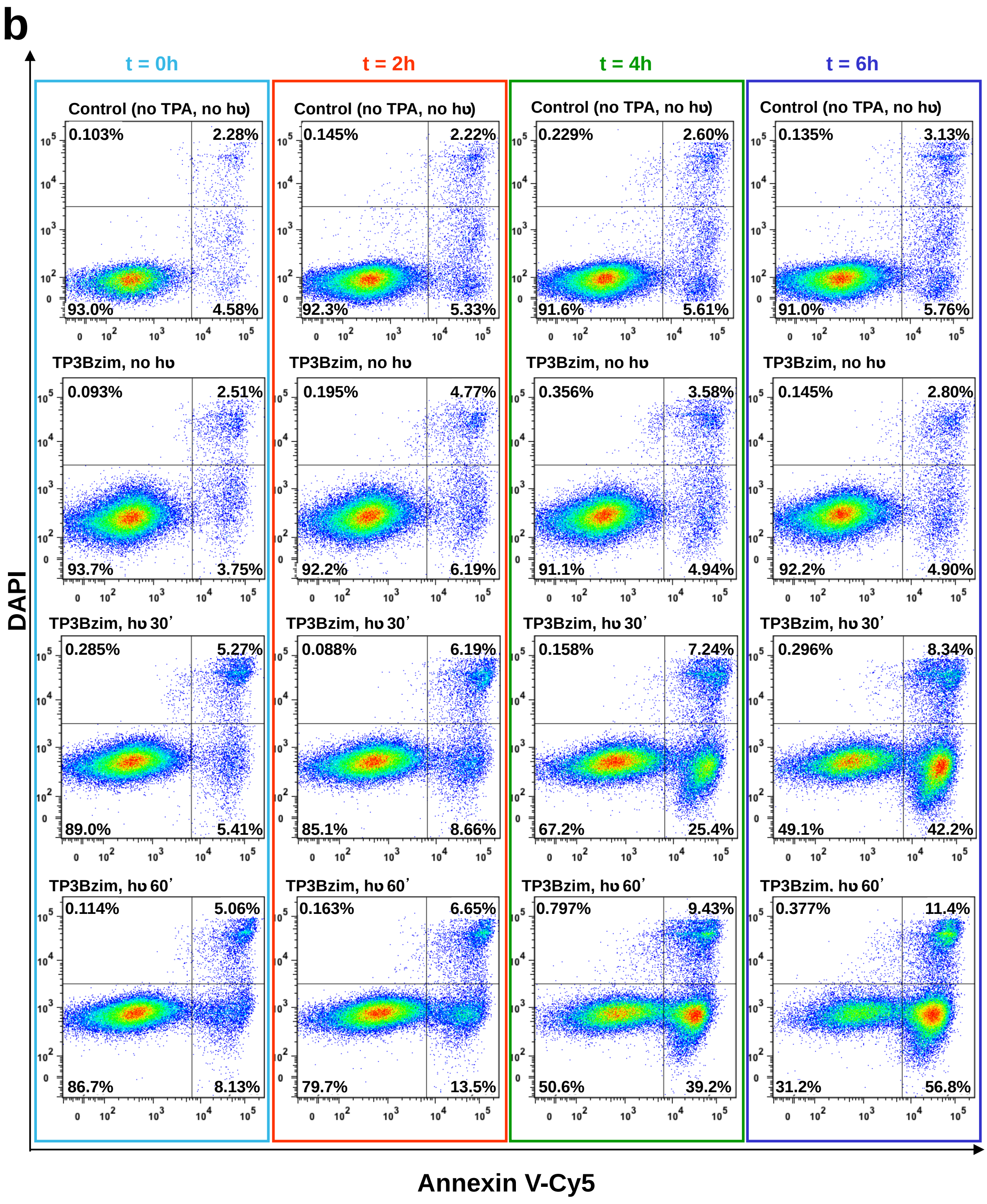

Figure S1. Flow cytometric analysis of Annexin V/DAPI staining of TPA-treated Jurkat cells. Jurkat cells were pre-incubated with 2 μM TP2Py (**a**) or TP3Bzim (**b**) for 2h in the dark at 37°C and subjected to light illumination for either 30 min (3rd line) or 60 min (4th line) at 452 nm (17 mW/cm^2). After light exposure, cells were either immediately (t=0; light blue) treated for Annexin V/DAPI staining or further incubated for various times in the dark at 37°C before Annexin V/DAPI treatment: **a**, t = 4h (red); t = 6h (green); t = 8h (dark blue); **b**, t = 2h (red); t = 4h (green); t = 6h (dark blue). 1st line: untreated control cells (without TPA, without light illumination). 2nd line: TPA-treated control cells (without light illumination). Annexin V was labelled with FITC (panel a) or Cy5 (panel b). The horizontal and vertical axes correspond to fluorescence intensities of Annexin V-FITC/Cy5 and DAPI, respectively. The 2-D dot plots shown in panels a & b are representative of three independent experiments.

Table S1: Times of illumination required for the observation of cellular events upon two-photon excitation of TP3Bzim at various wavelengths.

	Two-photon excitation of TP3Bzim[a]				
	**740 nm**	**760 nm**	**780 nm**	**800 nm**	**820 nm**
Appearance of blebs	3 min	2.5 min	3 min	5 min	10 min
Beginning of the nuclear translocation	6 min	6 min	6 min	6 min	13 min
Nuclear staining (max)	18 min	9 min	13 min	11 min	21 min

[a]Irradiance: 1.25 W/cm^2

Table S2: Times of illumination required for the observation of cellular events upon two-photon excitation of TP2Py at various wavelengths.

	Two-photon excitation of TP2Py[a]			
	**840 nm**	**860 nm**	**880 nm**	**900 nm**
Appearance of blebs	19.5 min	10 min	9 min	nd[b]
Beginning of the nuclear translocation	21 min	10 min	12 min	nd[b]
Nuclear staining (max)	34.5 min	20 min	24 min	nd[b]

[a]Irradiance: 1.25 W/cm^2. [b]nd: not determined due to photobleaching
